# Factors associated with the quality of death certification in Brazilian municipalities: A data-driven non-linear model

**DOI:** 10.1371/journal.pone.0290814

**Published:** 2023-08-31

**Authors:** Guilherme Augusto Zimeo Morais, João Luiz Miraglia, Bruno Zoca de Oliveira, Sóstenes Mistro, Wilian Hiroshi Hisatugu, Djeniffer Greffin, Clément Bernardo Marques, Eduardo Pontes Reis, Hugo Martins de Lima, Claudia Szlejf

**Affiliations:** 1 Department of Big Data, Hospital Israelita Albert Einstein, São Paulo, São Paulo, Brazil; 2 Multidisciplinary Institute of Health, Federal University of Bahia, Vitoria da Conquista, Bahia, Brazil; 3 Department of Computing and Electronics, Federal University of Espirito Santo, Vitoria, Espírito Santo, Brazil; 4 Sabin Diagnostic Medicine, Brasilia, Federal District, Brazil; 5 NeuralMed, São Paulo, São Paulo, Brazil; 6 Samel Group, Manaus, Amazonas, Brazil; Faculdade Sao Leopoldo Mandic, BRAZIL

## Abstract

Studies evaluating the local quality of death certification in Brazil focused on completeness of death reporting or inappropriate coding of causes of death, with few investigating missing data. We aimed to use missing and unexpected values in core topics to assess the quality of death certification in Brazilian municipalities, to evaluate its correlation with the percentage of garbage codes, and to employ a data-driven approach with non-linear models to investigate the association of the socioeconomic and health infrastructure context with quality of death statistics among municipalities. This retrospective study used data from the Mortality Information System (2010–2017), and municipal data regarding healthcare infrastructure, socioeconomic characteristics, and death rates. Quality of death certification was assessed by missing or unexpected values in the following core topics: dates of occurrence, registration, and birth, place of occurrence, certifier, sex, and marital status. Models were fit to classify municipalities according to the quality of death certification (poor quality defined as death records with missing or unexpected values in core topics ≥ 80%). Municipalities with poor quality of death certification (43.9%) presented larger populations, lower death rates, lower socioeconomic index, healthcare infrastructure with fewer beds and physicians, and higher proportion of public healthcare facilities. The correlation coefficients between quality of death certification assessed by missing or unexpected values and the proportion of garbage codes were weak (0.11–0.49), but stronger for municipalities with lower socioeconomic scores. The model that best fitted the data was the random forest classifier (ROC AUC = 0.76; precision-recall AUC = 0.78). This innovative way of assessing the quality of death certification could help quality improvement initiatives to include the correctness of essential fields, in addition to garbage coding or completeness of records, especially in municipalities with lower socioeconomic status where garbage coding and the correctness of core topics appear to be related issues.

## Introduction

Functioning civil registration and vital statistics (CRVS) systems provide comprehensive, timely, reliable, continuous, permanent, and up-to-date population and mortality statistics enabling governments to deliver health and social development programs more effectively. Despite their recognized importance for population health, progress in the quality of CRVS systems around the world has been slow, with many low-income and middle-income countries still lacking adequate mortality systems covering their whole population and registering reliable cause of death information. However, some low-income and middle-income countries have conducted CRVS improvement initiatives resulting in substantial progress [1, 2].

In Brazil, the Ministry of Health’s Mortality Information System (MIS) has been collecting and providing death statistics since 1975 [3]. In recent decades, the Brazilian Ministry of Health has implemented several strategies to upgrade the quality of death certification, including the improvement in data collection, regularity and flow [4], field investigation of ill-defined causes of death [5, 6], implementation of the National Network of Death Verification Service [7], suspension of federal funding to municipalities that do not comply with the expected standards [8], and education programs [9]. These actions have been successful in improving the reliability of MIS, represented by higher completeness of death reporting and the reduction in the registration of ill-defined causes of death throughout the country [3, 10–13]. Nevertheless, the country still has opportunities for improvement in the quality of death certification. Brazilian performance has remained below very-high performance countries in the vital statistics performance index [2], a single composite metric developed to assess CRVS performance based on completeness of death registration, quality of death reporting, quality of age and sex reporting, data availability, and timeliness [14].

Moreover, subnational disparities in the quality of death statistics are still of concern, with poorer performance in many states from the North and Northeast regions and municipalities in all regions [11–13, 15, 16]. Good quality subnational death statistics are essential for developing and implementing local health policies and previous studies have investigated local factors associated with completeness and reporting of ill-defined causes of death in Brazilian states and municipalities. Costa et al (2020) found that completeness of mortality data from the MIS and the civil registry were lower in municipalities in the lowest deciles of education and population density in 2015 and 2016 [12]. The rates of reported ill-defined causes of death between 1998 and 2012 were higher in states with a lower gross domestic product, higher social inequality, and higher rates of illiteracy [17]. Additionally, in Brazilian municipalities, the rates of ill-defined causes of death were found to be inversely associated with the size of the municipality [11, 18] and its per capita gross domestic product [18].

Although completeness and causes of death are key components to assess the quality of death reporting, additional information from death certificates is also relevant to produce accurate statistics. The United Nations recommends core topics to be investigated for death statistics purposes related to characteristics of the event and the decedent [19]. To the extent of our knowledge, few studies have investigated the poor quality of reporting measured by missing data in the Brazilian MIS database. Moreover, these works were restricted to specific regions, age groups, or death circumstances [18, 20–22]. Understanding the quality of death reporting based on missing or unexpected data in these core topics in Brazilian municipalities and whether local socioeconomic factors are associated with this quality performance would provide information that could be useful to achieve higher quality standards in death statistics. Therefore, we aimed to describe the quality of death reporting based on missing and unexpected values in core topics from death certification in the MIS database from 2010 to 2017. We also aimed to investigate whether this approach to assess the quality of death reporting was correlated with a more usual approach based on the reporting of causes of death that are not useful in the analyses of public health and mortality.

Towards the goal of assessing the association of municipality socioeconomic and health infrastructure context with the observed outcome, we employed a data-driven approach. Traditional linear models present strong assumptions about the underlying data, such as linear relationship between dependent and independent variables as well as the absence of multicollinearity. As socioeconomic variables in Brazilian municipalities are highly correlated [23], we chose to find the best model based on the data, by using a grid search technique to assess, in a cross-validation manner, which model would present the best performance. Finally, once the best model was chosen, and the performance was validated against unseen data, we aimed to assess the importance of the features for the model and to leverage the predictive modeling approach to identify outliers. In other words, we aimed to identify municipalities that, based on their socioeconomic and health context, would be expected to present a greater or worse quality of death certificates according to the model. By exploring the non-linear interactions between socioeconomic and health factors provided by the model, policymakers could have insights on patterns of such factors related to poor quality of death certification and use this information to guide interventions.

## Methods

This retrospective study was conducted mainly with data collected from the Brazilian Mortality Information System and included death certificates registered between 2010 and 2017. This is a disidentified and publicly available database administered by the federal government, therefore this study was not submitted for ethical approval. All analyses were performed in Python 3.7 and the Mortality Information System database can be accessed through this link: ftp://ftp.datasus.gov.br/dissemin/publicos/SIM/

### Municipal death certification quality

We assessed the quality of death certification based on the occurrence of missing or unexpected values on MIS variables that represent the core topics recommended by the United Nations to be investigated for death statistics purposes [19]. We included core topics for which information is collected directly and not derived (for example, age is considered a core topic, but it is derived from the date of birth and date of occurrence). We considered characteristics of the event (date of occurrence, date of registration, place of occurrence, and certifier) and characteristics of the decedent (date of birth, sex, and marital status) [19]. We excluded the following core topics: the place of registration as it was not available in the MIS database and cause of death because this variable was used in the correlation analyses. Values that differed from the MIS predefined codification list were considered as unexpected (for example: in the entry field for sex, the possible options are “male”, “female” or “ignored”. Anything different from these options was considered as unexpected). The distributions of the percentage of death certificates with any missing or unexpected values in any core topic and in each core topic among municipalities were visualized with box plots.

### Correlation between quality of death certification and garbage codes

Death certificates with missing or unexpected values in one or more core topics were considered of poor quality, and the total percentage of poor-quality certificates was calculated for each municipality. Also, we assessed the percentage of garbage coding in each municipality. Garbage codes refer to all deaths assigned to codes that should be redistributed to enhance the validity of public health analysis. We used the list of codes proposed by Naghavi et al, which was based on the Global Burden of Disease Study from 2010 [24]. This list includes the following types of garbage coding: deaths assigned to ICD codes that could not be underlying causes of death (e.g., senility or low back pain); that were intermediate causes of death rather than the underlying cause (e.g., sepsis and heart failure); that were immediate causes of death that are final steps in a disease pathway leading to death (e.g., disseminated intravascular coagulation syndrome); or that lacked specificity in coding (e.g., unspecified cancer or unspecified cardiovascular disease) [24]. Scatter plots and Spearman correlation coefficients were obtained to evaluate the correlation between the percentage of poor-quality death certificates assessed by missing or unexpected values on core topics and the percentage of garbage codes in the municipalities. Since the quality of death certification according to ill-defined causes has been associated with municipal socioeconomic factors in Brazil [10, 18], we stratified this analysis using GeoSES quartiles, a municipal index that summarizes the main dimensions of the Brazilian socioeconomic context for health and social research. The index ranges from -1 to 1 and it is composed of six dimensions: education, poverty, wealth, income, segregation, and deprivation [23]. This database is publicly available and can be accessed through this link: https://opendatasus.saude.gov.br/dataset/geoses. Inference with confidence intervals and hypothesis tests was not performed, given that the study included the whole “population” of death certificates and municipalities [25].

### Municipal demographic and socioeconomic characteristics

Municipal death rates (per 1000 population) included in the predictive models, were obtained by dividing the number of deaths reported in the MIS in 2010 by the number of inhabitants, which was extracted from the 2010 Demographic Census performed by the Brazilian Institute of Geography and Statistics. This database is publicly available and can be accessed through this link: http://www.atlasbrasil.org.br.

Socioeconomic municipal characteristics included in the predictive models (education, poverty, wealth, income, and deprivation) were derived from the GeoSES. For the present analyses, the values of the dimensions “education” and “deprivation” were multiplied by –1 to facilitate their interpretation, since to compose the index, “education” was explained by people without instruction, and “deprivation” was explained by car ownership [23].

### Municipal healthcare services infrastructure

Hypothesizing that health infrastructure could also be related to the quality of death certification, we collected information on municipal healthcare services infrastructure from the 2010 National Healthcare Facilities Registry (CNES). The CNES is a publicly available national database containing information on the installed capacity and health human resources of all Brazilian healthcare facilities, which can be accessed through this link: ftp://ftp.datasus.gov.br/dissemin/publicos/CNES/. The following municipal indicators were included in the predictive models: number of physicians/1000 population, number of hospital beds/1000 population, percentage of healthcare facilities that provided care through the Brazilian Universal Health System, and percentage of healthcare facilities that had emergency departments. The total population of each municipality was extracted from the 2010 Demographic Census.

### Predictive models

Classification models were built to predict the quality of death certification as the dichotomous target variable (good quality/poor quality), having municipalities as the units of observation. Predictive models were chosen to better fit the multivariate, non-linear data. To create the dichotomous target variable, municipalities were classified as having poor quality of death certification if the percentage of death certificates with missing or unexpected values (correct entries) for any core topic was ≥80%. This threshold was empirically defined based on the median percentage of all municipalities. The following steps, described in further detail below, were employed to build models, select and evaluate the model with the best performance: data splitting, grid search, assessment of metrics from multiple models, and assessment of features importance and residuals from the best performing model.

- Data splitting: The data was initially split into three different sets: 70% for training, 15% for validation, and 15% for testing. To avoid overfitting, the training set was further split within the grid search, which consisted of a 5-fold cross-validation iterator with non-overlapping groups. Data splitting respected the Brazilian division of health regions, therefore municipalities belonging to the same region were not separated [26]. This was done to avoid potential data leakage between sets due to spatial correlation, as neighboring municipalities were expected to have similar features.
- Grid search: A grid search technique was used to automatically split the data and assess a set of various linear and non-linear models. Linear models (logistic regression and stochastic gradient descent) and support vector machines were coupled with a standardization preprocessing step, while tree-based models (decision tree, random forest and gradient boosting machine) received the raw data directly. Hyperparameters used in each model can be seen in the S1 Table. To facilitate the assessment in a single run, the grid search was coupled with the scikit-learn pipeline. This also ensured that the preprocessor is fitted only on the training subset, and therefore avoided data leakage [27]. The grid search was set to obtain the highest area under the receiver operating characteristic (ROC) curve, and to refit the best estimator, once it was found, on the whole training set. To avoid overfitting, the validation set was used to assess the best estimator performance while the study was being developed, and the test set was used only once to generate the results and figures.
- Metrics: The main metric used to select the best model was the area under the ROC curve. In the context of predictive modeling, it provides an overview of the true positive and false positive rates with respect to the threshold probability chosen for the model. A value of 0.50 is used as a reference for a random estimator, while a perfect model would present a value of 1.0. The precision-recall curve, which assesses the true positive rate and the positive predictive value, was also obtained for the different model probabilities. Its reference value for a random model depends on the proportion of classes in the model, which resulted in a value of 0.47 for the final selected model. To further evaluate the model performance, histograms were used to explore the distribution of test samples per class and the predicted probability by the model. For a given probability threshold (e.g., 0.50) fixed for the model, the ratios of positive and negative outcomes were assessed toward the expected classification.
- Features importance: The Shapley Additive exPlanations (SHAP) was used to understand the contribution of each feature to the final predictive model, as it provides additional insight on the direction of the feature values and the respective impact on the model output [28].
- Residuals: The residuals of the model’s predictions were computed as the difference between the observed value (0 = good quality or 1 = poor quality) and the model predicted probability (between 0 and 1) for the poor quality of death certification. These estimates were used to identify municipalities with socioeconomic and health infrastructure characteristics that, according to the model, were incompatible with the quality of death certificates.

The scripts used to run the models and reproduce the results using the publicly available databases can be found in (https://github.com/GuilhermeZimeo/quality-death-cert).

## Results

**Fig 1:**
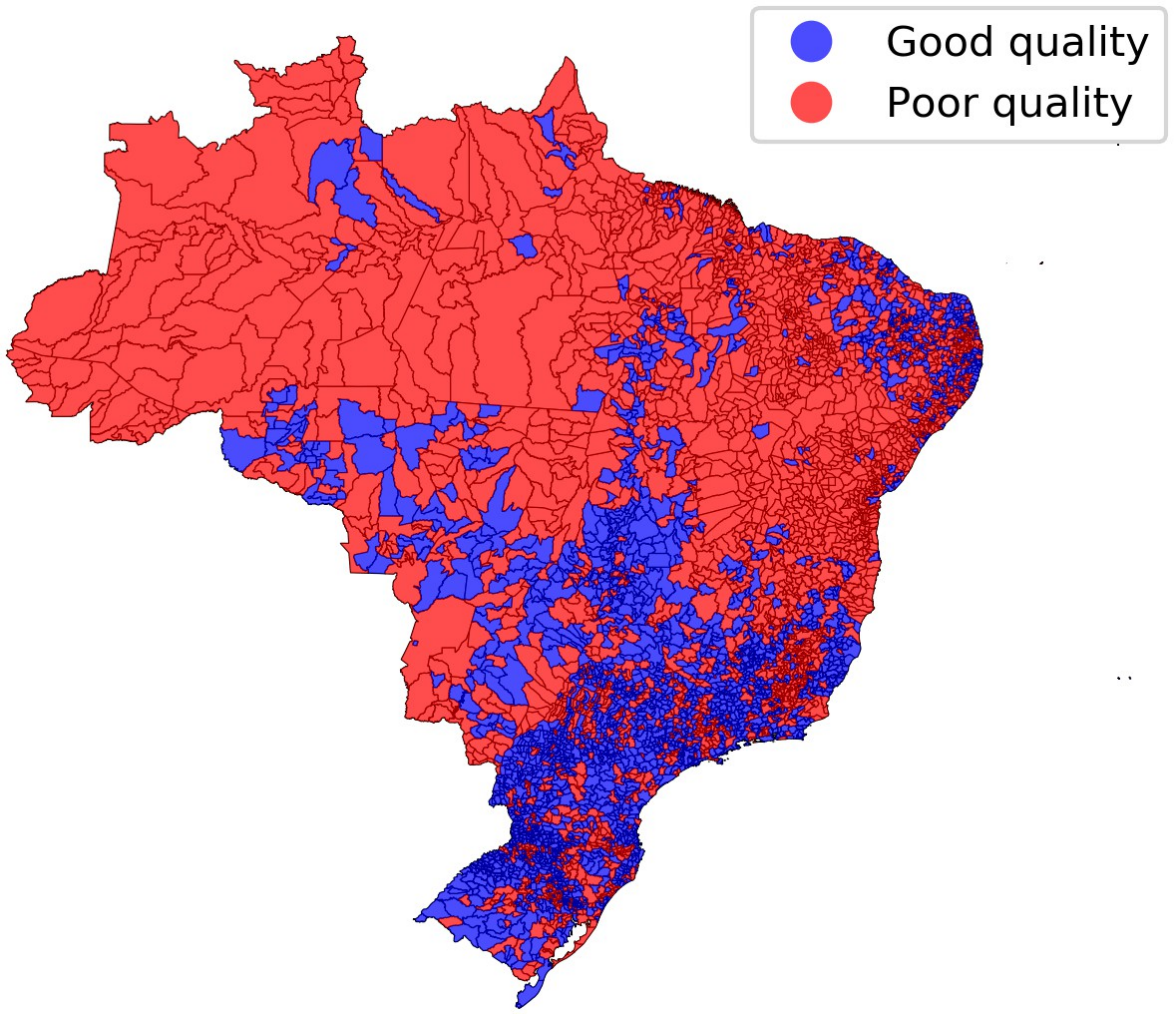
Distribution of quality on death certification per Brazilian municipality, between 2010 and 2017, based on missing or unexpected values on core topics.

The analyses included 9,812,520 death certificates, from 5570 municipalities with a median of 305 certificates per municipality (p25 = 122; p75 = 828). The distribution of death certification quality per municipality can be visualized in Fig 1, with 43.9% of municipalities presenting poor-quality certification, and a higher prevalence of municipalities with poor quality in the North (70.4%) and Northeast (70.9%) regions, when compared to the South (24.3%), Central-West (37.7%), and Southeast (38.5%) regions.

Municipalities with poor quality of death certification presented larger populations, lower death rates, lower scores for socioeconomic characteristics, and healthcare services infrastructure with fewer beds and physicians, with a higher proportion of healthcare facilities that provided care through the Brazilian Universal Health System, when compared to municipalities with good quality of death certification (Table 1), however, these distributions presented some overlap.

**Table 1:** Characteristics of municipalities with good and poor quality of death certification based on missing or unexpected values in core topics.

Characteristics	Good quality	Poor quality^a^
	median (interquartile range)	
Population	10,195 (4,679; 22,343)	12,578 (6,030; 24,481)
Education^b^	-66.0 (-71.9; -60.5)	-73.4 (-78.2; -67.0)
Poverty^b^	31.9 (20.7; 51.6)	60.5 (36.9; 70.9)
Wealth^b^	0.52 (0.21; 1.01)	0.33 (0.13; 0.69)
Income^b^	1,886.3 (1,389.4; 2,400.8)	1,220.4 (959.7; 1,800.0)
Deprivation^b^	-29.9 (-40.0; -14.5)	-10.1 (-24.7; -5.7)
Physicians (per1,000 population)	1.14 (0.77; 1.75)	0.83 (0.54; 1.31)
Hospital beds (per 1,000 population)	1.60 (0.00; 2.99)	1.38 (0.00; 2.50)
Facilities that provided care through the Brazilian Universal Health System (%)	84.6 (53.3; 100.0)	100.0 (72.2; 100.0)
Facilities with emergency department (%)	7.04 (0.00; 15.34)	7.69 (0.00; 14.29)
Death rate in 2010 (per 1,000 population)	3.60 (2.37; 5.08)	3.27 (2.29; 4.53)

^a^Poor quality defined if the percentage of death certificates without missing or unexpected values (correct entries) for any core topic was lower than 80%.

^b^Derived from GeoSES.

**Fig 2:**
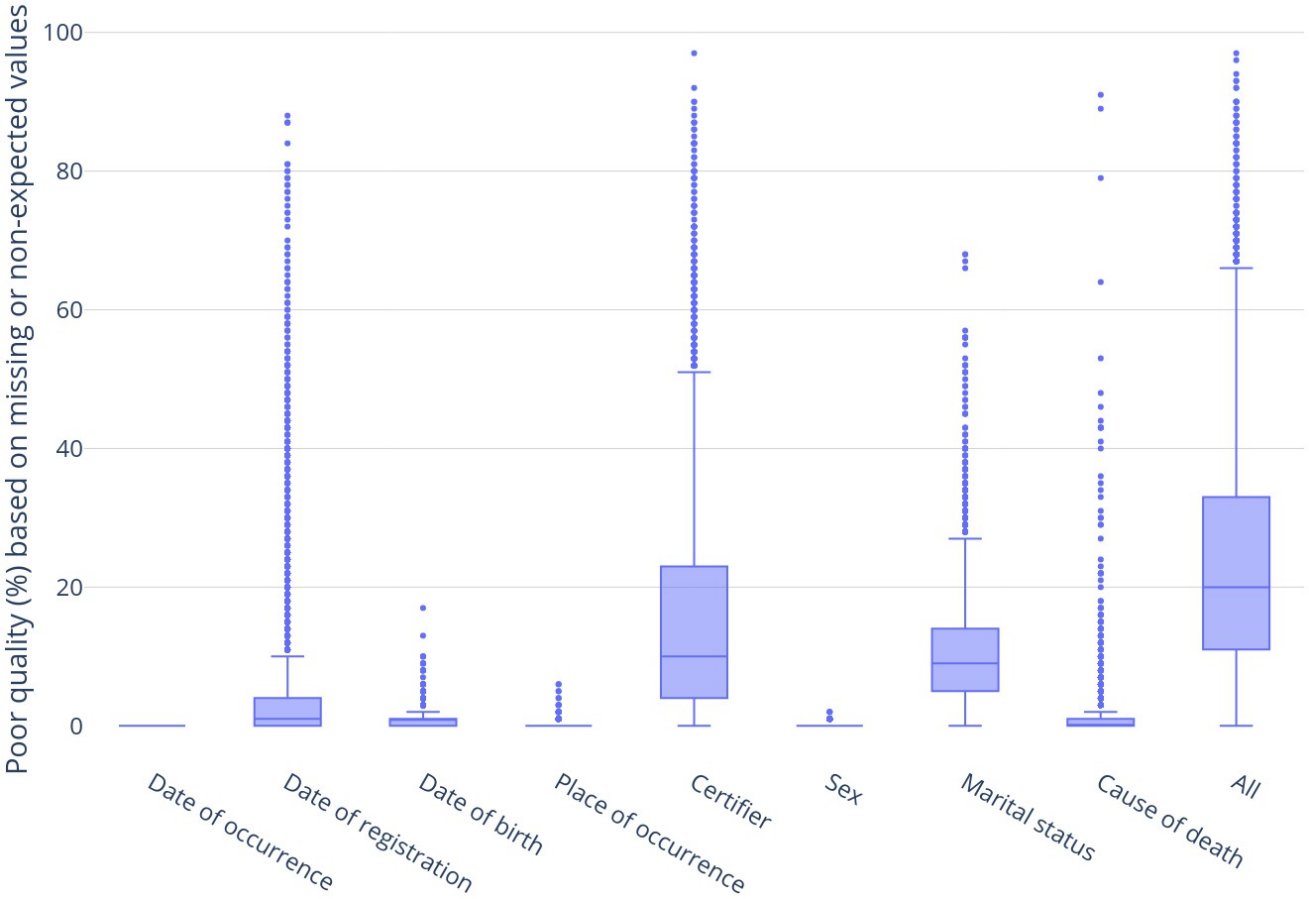
The distribution of death certificates with missing or unexpected values for each core topic among Brazilian municipalities.

The distribution of death certificates with missing or unexpected values for each core topic among municipalities can be found in Fig 2. Date of death, date of birth, place of occurrence, sex, and basic cause of death presented low percentages of missing or unexpected values for nearly every municipality, except for a few outliers. Date of registration, marital status, and certifier presented varying percentages of incorrect entries, with medians of 1%, 9%, and 10%, respectively.

**Fig 3:**
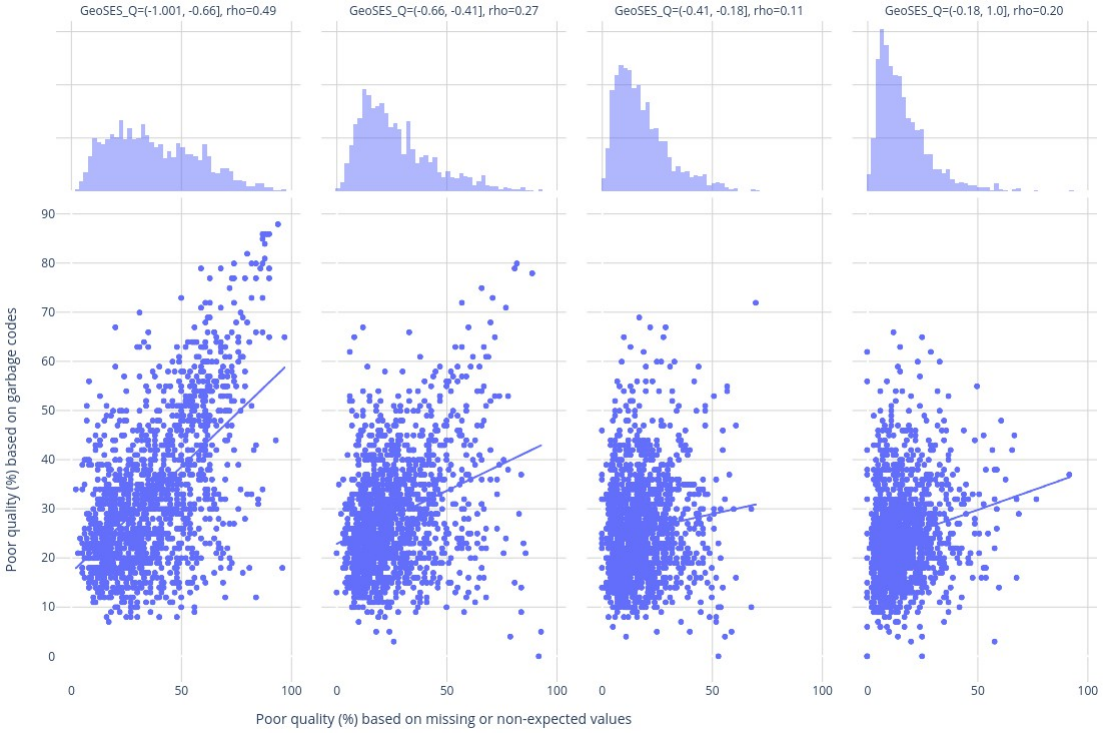
Distribution of metrics to assess poor quality of death certificates: Percentage of garbage codes (y-axis) vs percentage of missing/unexpected values in core topics (x-axis), and the respective Spearman correlation. Each point represents a Brazilian municipality. Subplots are stratified by GeoSES index quartiles (a municipal index that summarizes the main dimensions of the Brazilian socioeconomic context for health and social research) to better illustrate the distribution shift. Top: marginal distribution plot showing the distribution along the x-axis dimension. Bottom: scatter plot with trendline.

Overall, correlation coefficients between percentages of poor quality of death certification and percentages of garbage codes were weak, with a non-stratified coefficient of 0.33. In the stratified analyses (Fig 3), correlation coefficients ranged from 0.11 to 0.49, with a negative trend along with the GeoSES quartiles. It was also possible to identify in the histograms showing the marginal distribution of the percentage of poor quality of death certification, a clear progressive skewness towards poor quality percentages for lower GeoSES quartiles.

**Fig 4:**
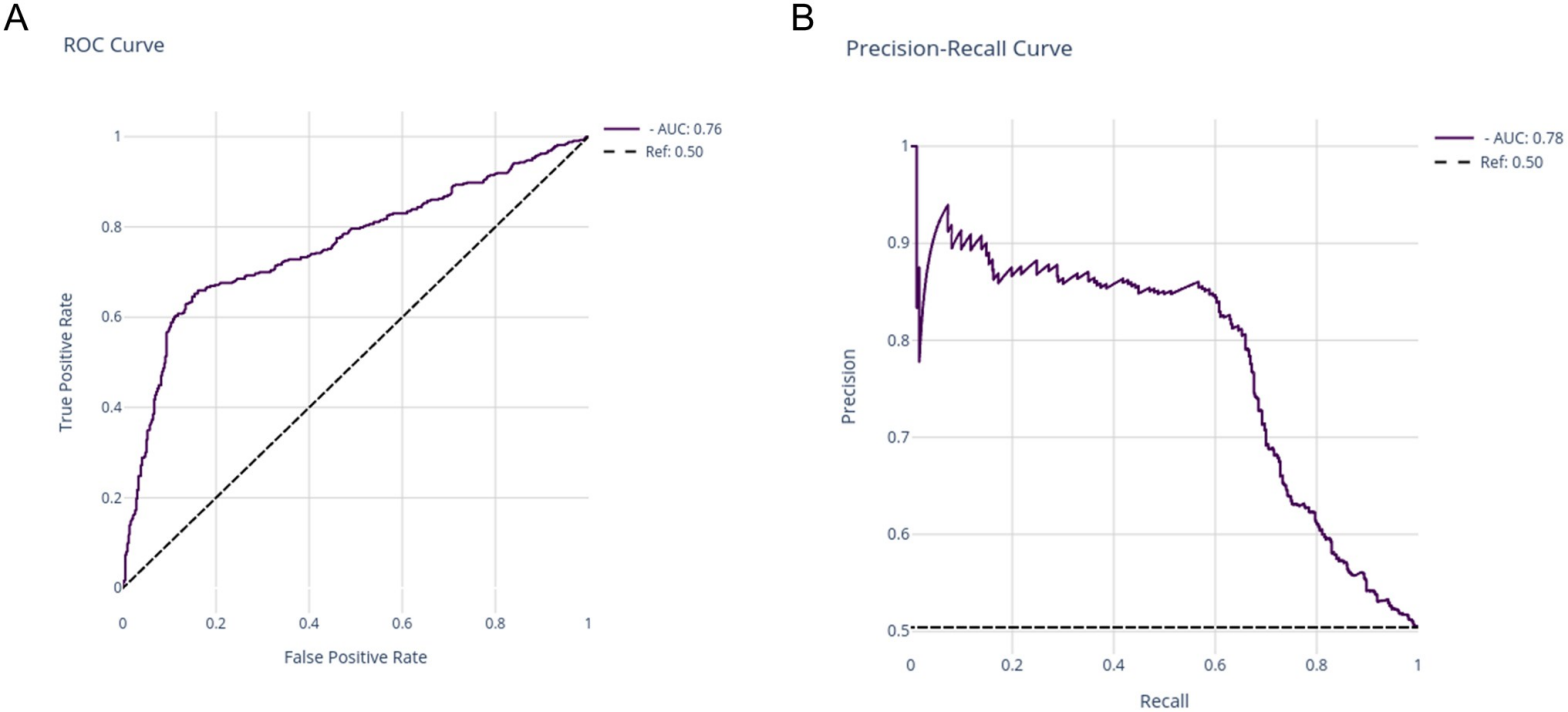
Receiver operating characteristic curve (left) and precision-recall curve (right) obtained from the random forest model applied to the test set. AUC, Area under the curve.

The predictive model returned from the grid search as the best estimator was the random forest classifier consisting of 1,000 decision trees, each of them with a maximum depth of 10. The details of all fitted models can be found in S1 Table. The test set ROC and precision-recall curves fitted with this model (Fig 4), showed acceptable discrimination (ROC AUC = 0.76; precision-recall AUC = 0.78).

**Fig 5:**
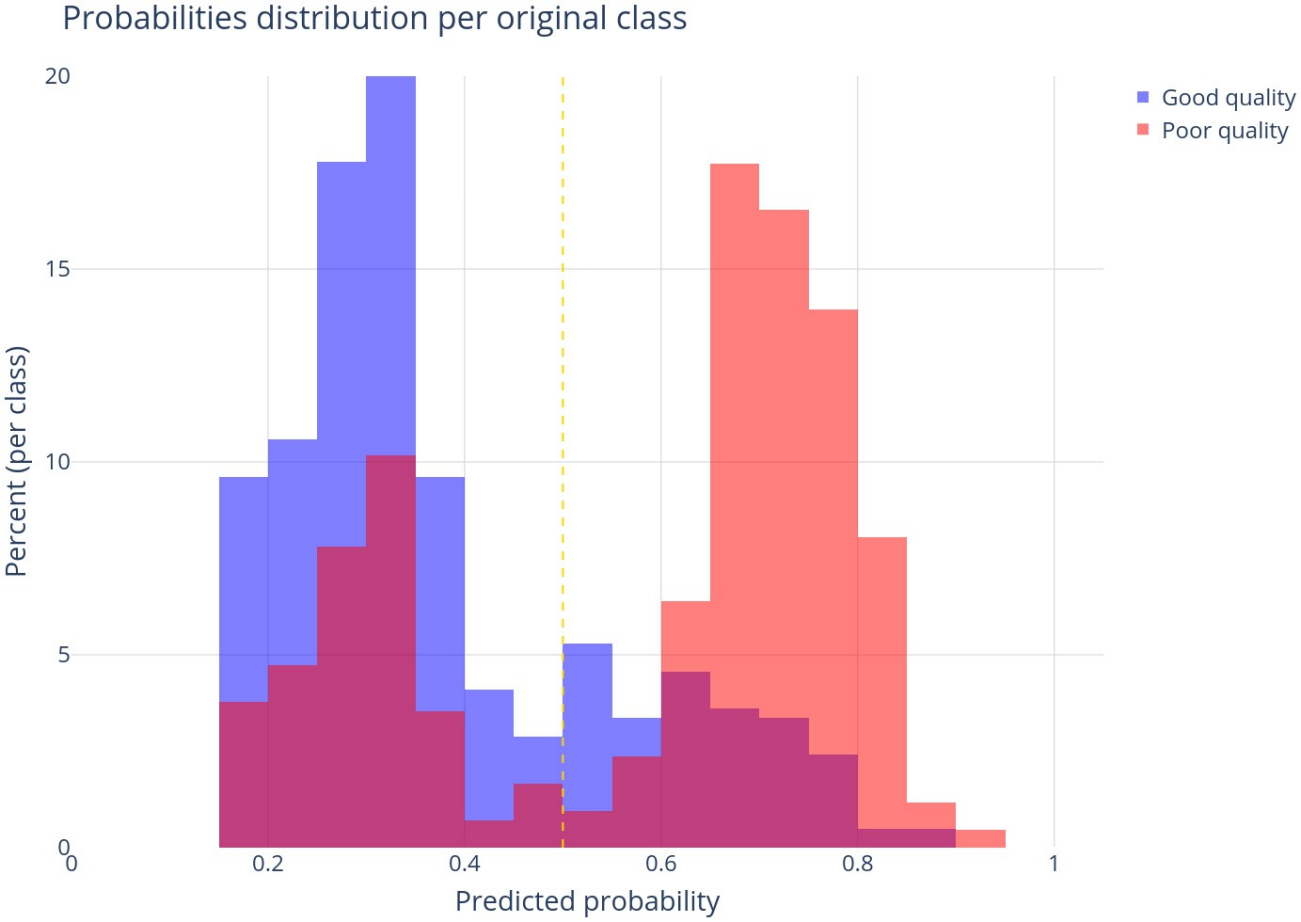
Distribution of tested municipalities in respect to the output probability of the model, according to the quality of death certification based on missing or unexpected values in core topics.

The performance of the random forest model for a fixed probability threshold of 0.50 can be visualized in Fig 5. For models with good performance, most samples labeled as “good quality of death certification” would be located on the left side of the vertical dashed line (probability < 0.5), and most samples labeled as “poor quality of death certification” would be located on the right side of the dashed vertical line (probability > 0.5). As the area under the ROC curve, these histograms show that the model has acceptable discrimination, resulting in a greater prevalence of true negative (76%) and true positive (67%) in respect to false positive (23%) and false-negative (32%) results.

**Fig 6:**
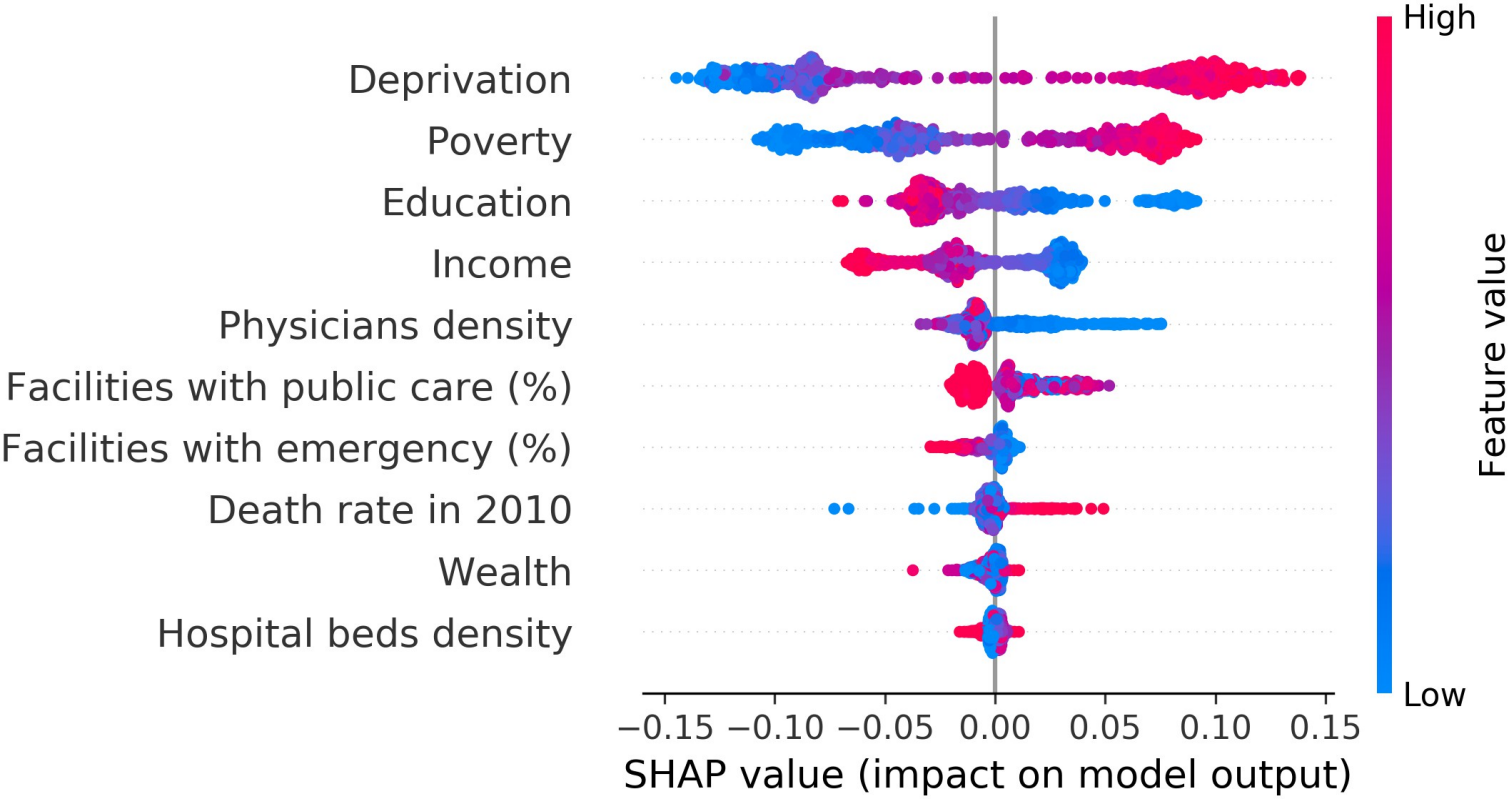
Impact of each feature available on the model output. The vertical axis lists the model features, and the horizontal axis measures the impact on classifying the municipal quality of death certification based on missing or unexpected values in core topics. The color represents the corresponding feature value (red stands for higher values of that feature, and blue lower values).

The SHAP results (Fig 6) showed that socioeconomic features were more important contributors to the prediction of quality of death certification than the health infrastructure of municipalities. Higher values of deprivation and poverty (in red) and lower values of education and income (in blue) were among the main contributors to the classification of a municipality as having poor quality of death certification.

**Fig 7:**
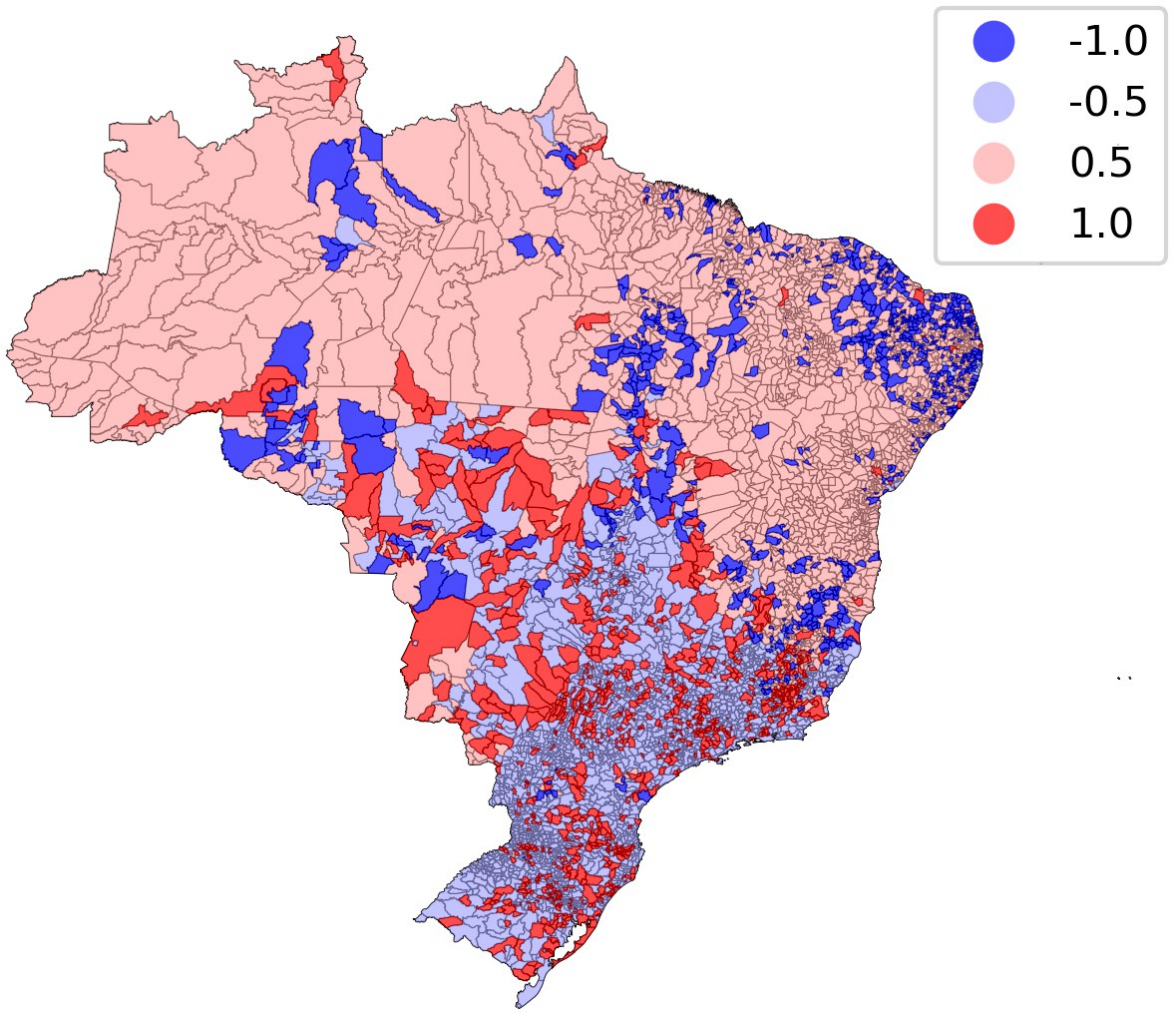
Map of model residuals per Brazilian municipality. Darker colors identify, according to the model, inconsistencies between the observed and the predicted outcome. Blue represents good quality and red represents poor quality of death certificates based on missing or unexpected values in core topics.

The distribution of the calculated residuals for the random forest model can be seen in Fig 7. Darker colors indicate municipalities with an inconsistency between observed and predicted quality of death certification. Municipalities depicted in dark blue presented good observed quality of death certification, even though their poor socioeconomic conditions and health infrastructure resulted in a poor predicted quality of death certification, representing 23% and 28% of municipalities in the North and Northeast regions, respectively. Conversely, municipalities depicted in dark red presented poor observed quality of death certification, even though their good socioeconomic conditions and health infrastructure resulted in a good predicted quality of death certification, representing 28%, 27%, and 24% of the municipalities in the Central-West, Southeast, and South regions, respectively.

## Discussion

CRVS systems should aim for the highest quality in terms of completeness, correctness, availability, and timeliness, which depends on the implementation of processes for quality assurance and assessment, including the measurement of entry correctness. More specifically, in order to target efforts to improve the utility of death certification data for policy, it is important to first recognize key errors, which has been mainly focused on the correctness of codification of causes of death. However, other entry fields are also considered as core topics and their evaluation, including the frequency of missing or unexpected values, could bring additional insights regarding the quality of death certification.

Since the 1980s, Brazil has registered a steady improvement in the quality of death certification, which was achieved by interventions aimed at increasing the completeness of death registration and improving cause-of-death certification [2]. In 1996, the proportion of deaths with ill-defined underlying causes (chapter 18 of ICD-10) was 15% in the country, with a small decrease until 2004 (reaching 12%). As of 2005, there was a sharp drop in this indicator, reaching 6% in 2015 [29]. This occurred because, in 2005, the Ministry of Health started a project to improve cause of death information, with a main focus on the North and Northeast regions. The main actions were: hiring supporters for the states; development of instruments for investigating deaths and mobile applications to subsidize and monitor municipalities in the process of investigating deaths with ill-defined causes; creation of the National Death Verification Services Network; conduction of record linkage between the MIS and other information systems; establishment of investigation goals and continuous monitoring of the results of the investigations and the indicator “percentage of deaths with ill-defined causes”; and cuts in federal funds if municipalities that did not reach the goal of information quality. In addition, as death certificate issuance is an integral part of medical care in Brazil, a new instruction manual for its filling was widely disseminated, to raise the awareness of physicians about the importance of the death certificate [30].

As of 2016, the Ministry of Health started to work with the concept of garbage codes to improve information quality on the underlying cause of death. This term refers to a concept introduced in the first Global Burden of Disease study. By definition, Garbage Codes are causes of death that would not be the underlying ones or would be unspecific, being, therefore, of little use for public health [24]. It is worth noting that this concept, in addition to encompassing ill-defined causes, still expands its scope, introducing well-defined codes that are not useful for designing intervention programs (such as sepsis or cardiac arrest, for example) [31]. More specifically, garbage codes are classified into four types: 1) Causes that cannot or should not be considered as a cause of death; 2) intermediate causes of deaths; 3) Immediate causes of death, which would be the last in the sequence of causes that led to death; 4) Non-specific causes within a large grouping of causes of death [24]. In the present study, we adopted the general concept of garbage codes, without considering their subtypes because we aimed to investigate whether two different approaches to investigate the quality of death certifications were correlated. Although we considered that assessing garbage coding subtypes would be outside the scope of the present analysis, we believe this can be an interesting advance for this study in future research.

Still, in 2016, the Ministry of Health joined the Data for Health Initiative, at the invitation of Bloomberg Philanthropies, which aimed to improve the lives of populations by improving the quality of information on mortality and using this information to formulate health policies. There were four priority interventions: to reduce the proportion of garbage codes in mortality statistics, through the investigation of deaths; to validate and introduce a reduced form for the application of verbal autopsy for deaths without medical assistance or with ill-defined causes of death; to develop a free mobile application in order to assist medical professionals in the correct death certification; to introduce the Iris software for coding the causes of death declared in the death certificate and automatic selection of the underlying cause in the MIS online module [6].

Despite these advances in the last decades, the quality of mortality data has been shown to vary among Brazilian regions and municipalities [1–13, 15, 16], with a similar finding in this study. In a study in 20 different countries, including Brazil, socio-demographic development was associated with the proportion of garbage codes [32]. Additionally, the authors observed that Brazilian municipalities with poor quality of death certification based on missing and unexpected values in core topics presented lower values of socioeconomic indicators, with nearly half of all municipalities fulfilling the criteria for poor quality of certification and the North and Northeast regions, the poorest in the country, presenting the highest percentages [32]. The discrepancy between country-wide quality and regional quality of death certification in Brazil is probably related to its known extremes in income and other social inequalities [33] and highlights the importance of more granular evaluations for policy decisions.

Sex and age were among the core topics that presented good quality for most municipalities and are the same variables that compose one of the dimensions of the vital statistics performance index [14]. An evaluation with data from 2015 found an index above 0.7 for Brazil, a level that is considered the cut-off for well-functioning systems [2]. However, this aggregated result does not capture the variability among municipalities and does not include the correctness of additional core topics that can impact the quality of death certification.

The correlation between percentages of poor quality of death certification and percentages of garbage codes was weak overall, but stronger for municipalities in the lowest GeoSES quartile, showing the possibility of effect modification on this association by socioeconomic status. This result could indicate that for municipalities with lower socioeconomic status, garbage coding and the correctness of core topics could be approached as a common issue, but for municipalities of higher socioeconomic status, they probably follow different pathways. Therefore, quality improvement initiatives focused on garbage coding could also impact the correctness of essential fields in municipalities with lower socioeconomic status. However, in municipalities with higher socioeconomic status, separated approaches would be warranted. On the other hand, the low correlation found could indicate that the basis associated with quality of death statistics measured by each approach differs. The relationship between different approaches to measure subnational quality of death statistics could be investigated in future studies, which should also include the assessment of completeness of death records. Targeted interventions to further improve death statistics could then be designed based on common factors.

The non-linear model showed acceptable discrimination and the socioeconomic variables derived from the GeoSES were the most important predictors of poor quality of death certification. Previous recommendations for quality improvement efforts of CRVS systems have been based on which components of the vital statistics performance index should be prioritized based on the country’s index [2]. The model here proposed could be used to target and evaluate complementary interventions focused on the improvement of the correctness of core topics. Finally, the use of the model residuals to identify municipalities with inconsistency between their actual and predicted quality of death certification could lead to new insights on the factors associated with poor quality of certification and possibly new quality improvement interventions.

It is important to mention the limitations of the present analysis. Although Brazil has been investing to increase the completeness of death records in the past decades [34], current regional disparities could have skewed the present findings, underestimating missing and unexpected values in regions with lower completeness. In addition, characteristics of the decedent, like young or old age, homelessness or extreme poverty, and death characteristics, like injuries, deaths that occurred outside health facilities, are also associated with poor death certification quality [35–40] and were not included in the predictive models. However, the present analysis focused on municipal characteristics, and including individual features in the models was outside its scope. Moreover, we were not able to include more up-to-date information on mortality and sociodemographic characteristics of municipalities. The most recent publicly available data on mortality was from 2017 due to the delay related to data validation performed by the Ministry of Health. Information on socioeconomic characteristics is collected every ten years in the demographic census and the most recent data was obtained in 2010. Finally, by using the median of the percentage of death certificates with missing or unexpected values to dichotomize the quality of death certification we were not able to model the original percentage of poor quality of death certificates among the municipalities. Nevertheless, we envision this choice has the potential to facilitate the communication of observed outcomes to national regional healthcare policymakers in more practical manners. The regional manager can identify a given municipality below the country’s average median and understand whether, according to our model and based on its socioeconomic and health infrastructure, such outcome is expected or not.

## Conclusions

This study investigated an innovative way to assess the quality of death certification through missing and unexpected values on core topics, which could be used as a complement to the more frequently employed, such as assessment of completeness, garbage coding evaluation or the composite vital statistics performance index on regions that have already reached good quality levels. Besides, a data-driven non-linear model with acceptable discrimination for this outcome was developed, which could be used to guide and evaluate quality interventions, while also pointing to new insights that could lead to new improvement policies.

## Supporting information

S1 Table Estimators, scaling, decomposition and parameters assessed within grid search. Table displaying grid search results for the machine learning models assessed.
(PDF)
