# Supplementary material for: Factors associated with the quality of death certification in Brazilian municipalities: A data-driven non-linear model
Source: PLoS One. 2023 Aug 31;18(8):e0290814. doi: 10.1371/journal.pone.0290814 (PMC10470916; doi:10.1371/journal.pone.0290814)
Supplement: S1 Table — Table displaying grid search results for the machine learning models assessed. (PDF) [file pone.0290814.s001.pdf]

Table S1. Estimators, scaling, decomposition and parameters assessed within grid search.

Estimator	Scaler	Decomposition	Parameters
Logistic Regression	Standard[a]	None	C: [0.001, 0.01, 0.1, 1, 10, 100, 1000]
Stochatisc Gradient Descent	Standard	None	l1_ratio: [0.1, 0.15, 0.2, 0.3]<br>alpha: [0.001, 0.01, 0.1, 1, 10, 100, 1000]
Support Vector Machine	Standard	PCA[b]	pca__n_components': [0.8, 0.9, 1]<br>kernel': ['rbf', 'linear']<br>C: [0.001, 0.01, 0.1, 1]
Decision Tree	None	None	max_features: [sqrt(n_features, n_features]<br>max_depth: [3, 5, 10, 20, 100]
Random Forest	None	None	n_estimators: [50, 100, 500, 1000]<br>max_depth: [3, 5, 10, 20, 100]
Gradient Boosting Machine	None	None	learning_rate: [0.0001, 0.001, 0.01]<br>colsample_bytree: [0.6, 0.8, 1.0]<br>n_estimators: [50, 100, 500, 1000]<br>reg_lambda: [0, 20, 50]

PCA: principal component analysis

[a]The “standard scaler” transforms the variables to have mean of 0 and standard deviation of 1.

[b]PCA that transforms the variables in orthogonal components.

All methods refer to the classes available in the scikit-learn library (version 0.22).
